# In silico and functional characterization of the promoter of a *Eucalyptus* secondary cell wall associated cellulose synthase gene (EgCesA1)

**DOI:** 10.1186/1753-6561-5-S7-P107

**Published:** 2011-09-13

**Authors:** Nicky Creux, Minique De Castro, Martin Ranik, Antanas Spokevicius, Gerd Bossinger, Christine Maritz-Olivier, Zander Myburg

**Affiliations:** 1 Department of Genetics, Forestry and Agricultural Biotechnology Institute (FABI), University of Pretoria, South Africa; 2 Agricultural Research Council Biotechnology Platform, Onderstepoort, South Africa; 3 Department of Forest Ecosystems and Society, Oregon State University, USA; 4 Department of Forest and Ecosystem Science, Melbourne School of Forest and Ecosystem Science, University of Melborne, Australia; 5 Department of Genetics, University of Pretoria, South Africa

## Background

Cellulose is an important biopolymer produced by all plants and is used in a number of different industries, including for pulp and paper production. Cellulose is deposited into the plant cell wall by a large membrane-bound protein complex, which is composed of different cellulose synthase (CESA) proteins. The cellulose content and pattern of deposition in plant cell walls is highly variable depending on the function of the cell. All plant cells have a thin primary cell wall, but a number of plant cell types, including xylem cells, also deposit a secondary cell wall to give these tissues mechanical strength required to perform their function. Different cellulose synthase (*CesA*) genes have been shown to be involved in the deposition of primary and secondary walls. In *Arabidopsis*, three *CesA* (*AtCesA4*, *7* and *8*) genes have consistently been associated with cells depositing secondary cell walls, while a different set of *CesA* genes have been shown to function during primary cell wall formation [Reviewed in 1]. These findings have been mirrored by studies of *CesA* gene orthologs in *Populus* and *Eucalyptus*[2-4]. While there have been a number of studies on *CesA* genes and their functions, much less is known about the regulation of these genes. In a previous study, we investigated the promoters of *CesA* genes involved in primary and secondary cell wall formation by performing a phylogenetic footprinting analysis to identify cis-elements conserved in the promoters from orthologous *Arabidopsis*, *Populus* and *Eucalyptus* cellulose synthase genes [5]. We identified a number of putative cis-regulatory elements that may play a role in the regulation of cellulose biosynthesis during primary and secondary cell wall formation. In the current study our aim is to further validate the cis-elements identified in the *CesA* gene promoters by investigating their conservation across different *Eucalyptus* species and to determine the regulatory function of these promoter regions and the proteins which bind to them.

## Methods

A number of different methods are being employed to investigate the regulatory functions associated with the *EucalyptusCesA* promoters. Firstly, to validate the cis-elements previously identified, we cloned and sequenced the promoters of six *CesA* genes from 13 different *Eucalyptus* species. The promoter sequences were analysed on the nucleotide diversity level. The cis-elements identified in the previous study were mapped onto the cloned promoter sequences and analysed for conservation in sequence and position. Next, we studied the possible roles of promoter regions harbouring conserved cis-elements in spatio-temporal regulation of *CesA* genes. We tested regions of the *EucalyptusgrandisCesA1* (*EgCesA1*) promoter for involvement in spatio-temporal regulation by cloning the full-length (2 kb) promoter and a series of truncates thereof upstream of the β-glucuronidase (GUS) reporter gene. These constructs were used to transform *Arabidopsis* (floral dipping) and *Eucalyptus* (Induced Somatic Sector Analysis, [6]). The GUS expression patterns were compared to the pattern produced by the 2 kb *EgCesA1* promoter. Finally, promoter regions identified as functionally active and harbouring conserved elements of interest are being used to screen a *Eucalyptus* immature xylem cDNA expression library for yeast-1-hybrid interactions to identify proteins which interact with these regions.

## Results and discussion

Studying the diversity of *CesA* promoter sequences and cis-elements in 13 *Eucalyptus* species provided us with valuable insight into the relative conservation of specific promoters regions and the cis-elements within these regions. We found that the overall nucleotide diversity of the promoter sets varied greatly from promoter to promoter, but we could identify regions in the promoters that were as conserved as coding regions. We found that in many cases these localized decreases in nucleotide diversity corresponded to clusters of conserved cis-elements which were identified previously [5]. This was particularly noticeable at the transcriptional start site for most genes and in this region we noticed a repeat element in all of the promoters investigated. Some elements were also shown to be specific to either the primary or secondary cell wall associated promoters. The cis-element information obtained from this study was used to create seven truncates of the *EgCesA1* promoter. Using GUS expression analysis in *Arabidopsis* we identified a number of repression and activation sites within the promoter. We also observed a loss of leaf (vein) expression 800 bp upstream. One of the repeat elements fused to the 5’UTR greatly enhanced overall GUS expression in a non-specific way. In a *Eucalyptus* background (ISSA), six of the seven truncates showed xylem-specific expression, but the 5’UTR and repeat element fusion showed GUS expression in phloem and xylem. These regions have been used to construct bait vectors for Yeast-1-hybrid screening which is still ongoing.

## Conclusion

In this study we addressed three main objectives, (1) investigate the evolution of the *EucalyptusCesA* promoters and cis-elements associated with primary and secondary cell wall formation, (2) investigate the expression patterns of truncated versions of the *EgCesA1* promoter using the GUS reporter system and (3) test the *EgCesA1* promoter regions affecting gene expression in a yeast-1-hybrid assay to identify possible regulators of this gene. We have identified regions in the promoter that were conserved and corresponded to previously identified cis-elements. Using this information we produced seven promoter truncates and discovered several regions and cis-elements in the *EgCesA1* promoter which affect GUS expression patterns. These results will aid in understanding and elucidating transcriptional networks regulating xylogenesis in woody genera such as *Eucalyptus*.
